# Analysis of *CDR1* and *MDR1* Gene Expression and *ERG11* Substitutions in Clinical *Candida tropicalis* Isolates from Alexandria, Egypt

**DOI:** 10.1007/s42770-023-01106-y

**Published:** 2023-08-22

**Authors:** Mohammed A. El-Kholy, Ghada F. Helaly, Ebtisam F. El Ghazzawi, Gamal El-Sawaf, Sherine M. Shawky

**Affiliations:** 1 Department of Microbiology and Biotechnology, Division of Clinical and Biological Sciences, College of Pharmacy, Arab Academy for Science, Technology and Maritime Transport (AASTMT), Alexandria, Egypt; 2 Department of Microbiology, Medical Research Institute, Alexandria University, Alexandria, Egypt

**Keywords:** *Candida tropicalis*, Azole resistance, Gene expression, Gene sequencing, Efflux pump, *CDR1*, *MDR1*, *ERG11*

## Abstract

**Introduction:**

*Candida tropicalis* is a common non-*albicans Candida* (NAC) species that causes numerous fungal infections. Increasing antifungal resistance to azoles in NAC is becoming a major health problem worldwide; however, in Egypt, almost no data is available regarding fluconazole resistance mechanisms in *C. tropicalis*. The current study aims to investigate two possible important molecular mechanisms involved in fluconazole resistance in *C. tropicalis* isolates.

**Materials:**

Fifty-four clinical *C. tropicalis* isolates were included. Identification and antifungal susceptibility profiles of the isolates were carried out using the VITEK 2 compact system. The molecular investigation of fluconazole resistance included the expression of the *CDR1* and *MDR1* genes by quantitative real-time RT-PCR as well as the sequence analysis of the *ERG11* gene.

**Results:**

Antifungal susceptibility testing identified 30 fluconazole-non-susceptible isolates. Statistically, *CDR1* gene expression in fluconazole-non-susceptible isolates was significantly higher than that in fluconazole-susceptible isolates, with *MDR1* gene expression levels that were similar in both non-susceptible and susceptible isolates. Sequence analysis of the *ERG11* gene of 26 fluconazole-resistant isolates identified two missense mutations: A395T (Y132F) and G1390A (G464S).

**Conclusions:**

This study has highlighted the role of overexpression of the *CDR1* gene and *ERG11* gene mutations in fluconazole non-susceptibility. Further studies in Egypt are required to investigate other possible molecular mechanisms involved in azole resistance.

**Supplementary Information:**

The online version contains supplementary material available at 10.1007/s42770-023-01106-y.

## Introduction

Candidiasis represents one of the most common causes of invasive fungal infections and is associated with high morbidity and mortality rates, especially in immunocompromised patients [1–3]. In the past decades, changes in the epidemiology of *Candida* infections have been noted. Although *Candida albicans* remains the most common isolated species in patients with invasive candidemia, a shift towards non-*Candida albicans Candida* (NCAC) species has been noted [4, 5]. *Candida tropicalis* has become a predominant NCAC species, causing invasive candidiasis [3, 6]. Currently, *C. tropicalis* ranks as the first or second NCAC species causing candidemia and candiduria according to the geographical region, such as East Asia and Latin America [5, 7–9]. After *C. albicans*, *C. tropicalis* is considered to be the second most virulent *Candida* species [10]. Patients infected with *C*. *tropicalis* usually have longer hospitalizations and higher mortality rates in comparison to those infected with *C. albicans* [6].

Among the different classes of antifungal agents used for the management of fungal infections, azoles are the most commonly used drugs for both treatment and prophylaxis. Azoles act through inhibition of lanosterol C14α-demethylase (Erg11p), which is the main enzyme involved in the ergosterol biosynthesis pathway. Erg11p is encoded by the gene *ERG11*. Fluconazole represents one of the most widely used antifungal agents, as it has minimal side effects in addition to its affordable cost compared to other classes with higher costs, such as echinocandins [11, 12].

Studies have reported a significant increase in azole resistance among *C. tropicalis* isolates, which could be attributed to the wide use of azoles as agents of antifungal prophylaxis and their widespread use in agriculture. This leaves a limited number of treatment options, endangering patients, especially in developing countries with limited resources [13, 14].

There are numerous molecular mechanisms that could lead to azole resistance. Alterations and/or overexpression of the lanosterol C14α-demethylase (*ERG11*) gene, upregulation of efflux transporter genes, the major facilitator superfamily (MFS) family gene (*MDR1*), and the ATP-binding cassette (ABC) transporter family (*CDR1*) are some of the most common molecular resistance mechanisms reported. Also, mutations in either the sterol D5,6-desaturase (*ERG3*) gene or the acquisition of functional mutations and/or overexpression of different transcription factors are involved in azole resistance [14, 15].

The current knowledge of azole resistance in *C. tropicalis* isolated from Egyptian hospitals is very scanty. Our study aimed to investigate some of the different molecular mechanisms involved in azole non-susceptibility in clinical *C. tropicalis* isolates.

## Material and methods

### Clinical isolates

A total of 54 *C. tropicalis* species clinical isolates were included from a previous study [16]. The clinical specimens were collected from various ICU patients admitted to different medical facilities, including the Medical Research Institute (MRI), Alexandria Main University Hospital (AMUH), and Mabaret Al-Asafra Hospital. Identification of isolates was carried out by the CHROMagar Candida and VITEK 2 compact system. The antifungal susceptibility testing of the isolates was determined using the VITEK 2 system AST-YS07 card. Out of the included isolates, 30 samples were fluconazole-non-susceptible (4 isolates were susceptible-dose-dependent (SDD) and 26 isolates were fluconazole-resistant), and 24 samples were susceptible to fluconazole. Regarding voriconazole, 28 isolates were non-susceptible (23 isolates were SDD and 5 isolates were voriconazole-resistant), and 26 isolates were susceptible to voriconazole. The interpretation of MIC readings was according to the CLSI species-specific clinical breakpoints (SS-CBPs) (CLSI M27-S4) for fluconazole, voriconazole, caspofungin, and micafungin [17], and the epidemiological cut-off values (ECVs) for flucytosine and amphotericin B [18]. (Supplementary Table 1) All isolates were stored as frozen stocks with 30% glycerol in a deep freezer at −20°C until used.

## Expression analysis of *CDR1* and *MDR1* genes by quantitative real-time RT-PCR (RT-qPCR)

All *C. tropicalis* clinical isolates were cultured on SDA for 48 hours. RNA extraction and purification for all the isolates were performed from the grown fungal cells using the YeaStar™ RNA Kit (Zymo Research Corp., USA), according to the manufacturer’s instructions. Samples were treated with RNAse-free DNAse (Qiagen, Hilden, Germany). Extracted RNA concentrations and purities were calculated using a NanoDrop 2000 spectrophotometer (Thermo Scientific) for the studied samples.

Samples were analyzed for relative expression of *CDR1* and *MDR1* genes using the SensiFAST SYBR® Hi-ROX One-Step kit (Bioline, UK), where cDNA synthesis and PCR amplification were carried out in the same tube using a fixed concentration of RNA template (0.1 μg/μL) for each sample. The protocol was followed according to the manufacturer's instructions. All the primers used for real-time RT-PCR were designed (Table 1) using the primer-BLAST (primer3) at www.ncbi.nlm.nih.gov, based on the *CDR1*, *MDR1,* and *Actin* (internal control) genes sequences of *C. tropicalis* MYA-3404 (GenBank accession no. GG692395) [19].

**Table 1:** Primers used in qRT-PCR and *Erg11* gene amplification

Primer	Sequence (5’–3’)	Amplicon length (bp)	Reference
Primers used in qRT-PCR			
*cdr1*-F	CAATCACATTCGTCCTGGTTC	387	This Study
*cdr*1-R	TTGAAAGCCAAGGACATCAC		
*mdr*1-F	ATGTTGGCATTCACCCTTC	426	
*mdr*1-R	GAAAACTTCTGGGAAAACTGG		
*actin*-F	AGCCGATTTAGGTTTGGAAG	325	
*actin*-R	GTGGTGGACAATAGATGGAC		
Primers used in *Erg11* gene amplification and sequencing			
*Erg11*-F	CACAGTTATAGACCCACAAGG	1789	This Study
*Erg11*-R	TACTTAGCAACAACTTCTAGTG		

Amplification of target genes was carried out using the StepOne ™ System (Applied Biosystems, USA) according to the following thermal profile: cDNA synthesis: one cycle at 45°C for 10 min, Polymerase activation: one cycle at 95°C for 2 min, followed by 40 cycles of amplification: Denaturation at 95°C for 5 sec, annealing at 55.5°C for 5 sec, and extension at 72°C for 5 sec. A melt curve analysis was performed at the end of the PCR cycles, confirming the specificity of the reaction and ensuring that only a single PCR product was amplified. (Supplementary figures 1–3).

Statistical analysis of gene expression was carried out using a one-sample t-test where appropriate by the Statistics Package for Social Sciences (SPSS) software version 21; a *p* value less than 0.05 (95% confidence interval of the difference) was considered significant.

## Sequencing of the *ERG11* gene and mutation analysis

Genomic DNA extraction and purification from the 26 fluconazole-resistant isolates were performed using the YeaStar Genomic DNA Kit™ (Zymo Research Corp.) according to the manufacturer's instructions. Extracted DNA concentrations and purities were checked using a NanoDrop 2000 spectrophotometer (Thermo Scientific, USA).

Primers were designed using the primer-BLAST (primer3) at www.ncbi.nlm.nih.gov, based on the *Erg11* gene sequence of *C. tropicalis* ATCC 750 (GenBank accession no. M23673) (Table 1). *Erg11* gene amplification was performed using the MyTaq™ HS Mix Kit (Bioline, UK). Amplification of target genes was carried out using the Veriti® Thermal Cycler (Applied Biosystem, CA, USA) according to the following thermal profile: Initial denaturation at 95°C for 1 min, followed by 35 cycles: Denaturation at 95°C for 15 sec, annealing at 55°C for 15 sec, and extension at 72°C for 10 sec. PCR products were purified using a PureLink® PCR purification kit (Invitrogen) according to the manufacturer's instructions.

### Sequencing and Data Analysis:

Sequencing of both strands was performed, and the nucleotide sequences obtained were analyzed using the BioEdit® sequence alignment editor and analysis program. Raw DNA chromatograms were visually checked and scrutinized for heterozygosity, which was defined as the presence of overlapping peaks in the forward and reverse chromatograms, and compared with *C. tropicalis* ATCC 750 (GenBank accession no. M23673) *Erg11* gene sequence. (Supplementary figures 4–9).

## Results

### Expression analysis of *CDR1* and *MDR1* genes by quantitative real-time RT-PCR (RT-qPCR)

**Fig. 1:**
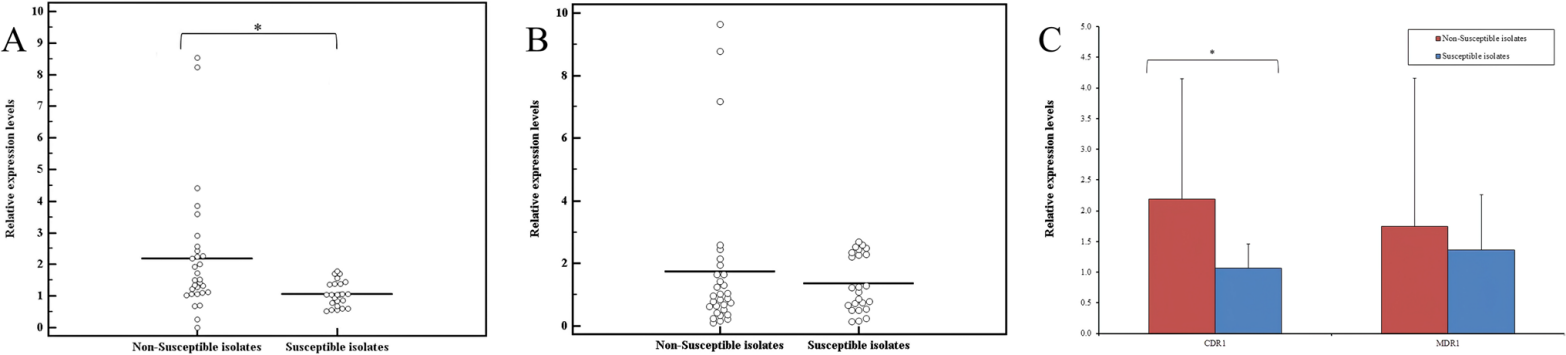
Relative gene expression of (**A**)* CDR1*, (**B**)* MDR1* efflux pumps genes, and comparison between the two groups (**C**) according to *CDR1* and *MDR1* relative gene expression levels in fluconazole-non-susceptible and susceptible *C. tropicalis* clinical isolates. * P ≤ 0.05

RT-qPCR showed that the relative *CDR1* gene expression in fluconazole-non-susceptible isolates was significantly higher compared to fluconazole susceptible isolates (average fold expression level 2.19 vs. 1.07, *p* < 0.01). However, no statistical difference was observed regarding the relative *MDR1* gene expression in fluconazole-non-susceptible isolates when compared to fluconazole-susceptible isolates (*p* = 0.589). (Figure 1). (Supplementary Tables 2–5)

## Sequencing of the *ERG11* gene and mutation analysis

The analysis of the chromatograms of the 26 fluconazole-resistant isolates revealed seven different mutations, two of which were missense mutations: A395T (Y132F), observed in only one clinical isolate, and G1390A (G464S), detected in 24 clinical isolates, while the other five mutations identified in all the tested isolates were silent mutations: T225C, G264A, G1362A, C1464T, and T1554C. (Table 2).

**Table 2:** The *ERG11* gene substitutions in fluconazole-resistant *C. tropicalis* isolates

Mutation site in the *Erg11* gene	No. of isolates showing mutation	Mutation type	Amino acid change
T225C	1	Silent	C75C
G264A	1	Silent	L88L
A395T	1	Missense	Y132F
G1362A	25	Silent	K454K
G1390A	24	Missense	G464S
C1464T	1	Silent	I488I
T1554C	25	Silent	I518I

## Discussion

Resistance to azoles, particularly fluconazole, is rising worldwide [20, 21]. In Egypt, limited data is available regarding the underlying mechanisms of azole resistance in *C. tropicalis*. In this study, we aimed to investigate some of the possible azole resistance mechanisms in *C. tropicalis* as mutations in the *ERG11* gene as well as the upregulation of the major facilitator gene *MDR1* and the ATP-binding cassette transporter gene *CDR1*.

Different studies reported various profiles of *CDR1* and *MDR1* gene expression. The current study investigated the role of efflux pumps in azole resistance by comparing quantitative relative gene expression for *CDR1* and *MDR1* genes in both fluconazole-susceptible and non-susceptible isolates. Our results showed that relative *CDR1* gene expression in fluconazole-non-susceptible isolates was statistically and significantly higher than that of susceptible isolates. On the other hand, relative *MDR1* gene expression in fluconazole-non-susceptible isolates was not statistically or significantly greater than susceptible isolates.

In agreement with our finding, Vandeputte et al. [22] stated that no difference was found in expression of the *MDR1* gene between fluconazole-susceptible and resistant isolates. This finding could be attributed to the fact that *MDR1* genes encode an MFS transporter, with a relative specificity for fluconazole. Thus, it is unlikely that *MDR1* gene overexpression could alter fluconazole susceptibility pattern in comparison to the *CDR1* gene, which encodes ABC transporters and is responsible for azole cross-resistance [23]. On the contrary, You et al. and Jin et al. found overexpression of the *MDR1* gene expression level in contrast to the *CDR1* gene, which showed no difference in gene expression [24, 25].

Although overexpression of both the *CDR1* and *MDR1* efflux pump genes was demonstrated in *C. tropicalis* isolates, highlighting that efflux pumps play a significant role in azole resistance in many studies [26–30], opposing results were reported in other studies demonstrating that efflux pumps do not play a vital role in azole resistance in *C. tropicalis*, as the *CDR1* and *MDR1* gene expression levels did not significantly differ between azole susceptible and resistant isolates [29, 31]. The inconsistencies and discrepancies in the literature's gene expression analysis may be attributed to different factors. There is an interplay of different resistance mechanisms, including ones that were not investigated. Overexpression of efflux pumps seems not to be the main azole resistance mechanism, as the EGR11 missense point mutation is the primary one, yet it contributes to the overall resistance. Some researchers observed that *C. tropicalis* isolates only had overexpression of a single gene target. Moreover, the difference may be caused by heterogeneity between the isolates. Other factors that may be considered are the number of included isolates, the MIC levels of fluconazole and other azole members of the tested isolates, reflecting the degree of cross-resistance between azole members, and the overall molecular mechanisms contributing to the azole resistance of each isolate [29–31].

Several studies have investigated *ERG11* gene mutations as one of the possible mechanisms for azole resistance in *C. tropicalis*. In the present study, sequencing analysis of the *ERG*11 gene in 26 fluconazole-resistant isolates showed that seven different mutations took place. Two missense mutations were detected leading to amino acid substitutions: A395T (Y132F) and G1390A (G464S), which were located in *ERG11* hot spots I and III, respectively, as previously described [32]. The other five mutations were silent mutations: T225C, G264A, G1362A, C1464T, and T1554C.

The G1390A (G464S) mutation was the most frequent type of mutation identified in our study. This amino acid substitution was also reported in other studies [28, 29]. Another amino acid substitution at the same site was also reported [33]. The A395T (Y132F) mutation may lead to decreased binding affinity for azoles [22]. However, it was only demonstrated in one isolate in our research; the A395T (Y132F) mutation was frequently observed in other previous studies [22, 30, 31, 33, 34].

Additionally, silent mutations have been demonstrated in our study, including G1362A and T1554C in 25 isolates (96.15%). However, these mutations have been reported previously in other studies; being synonymous mutations, they are usually considered silent and cannot therefore be linked to azole resistance [22, 33, 35]. It is worth mentioning that the percentage of silent mutations in relation to the total number of mutations detected in the current study was in agreement with a Chinese study conducted by Fan et al. (71.8%) [29]. Our study revealed the first reported silent mutation, C1464T (I488I). Although the role of synonymous mutations in azole resistance is not established, different reports have begun to unravel the role of synonymous mutations in altering the nucleic acid sequence and consequently its structure. synonymous mutations may affect RNA secondary structure, stability, and regulatory elements such as ribosome binding sites or splicing signals, giving rise to changes at the gene expression levels as well as substrate specificity, rendering them not entirely silent. Furthermore, synonymous mutations can affect codon usage and the accuracy and speed of translation. Changes in codon usage may reflect on protein expression levels, stability, and folding, which can indirectly influence resistance mechanisms. Studies have revealed that the presence of synonymous codons is non-random and may participate in antimicrobial resistance [36–40]. According to our best knowledge, the various possible effects of synonymous mutations have not been described or investigated in clinically relevant fungi. Further studies are warranted to investigate any possible mechanisms.

The main limitation of the current study, due to financial restrictions, was the inability to investigate the role of other molecular mechanisms of azole resistance. For instance, the *ERG11* gene expression in addition to the sequence analysis of efflux pumps genes *CDR1* and *MDR1* are examples of these limitations. Furthermore, the role of *UPC2* (expression regulator) and *ERG3* mutations in azole resistance, as well as gene expression, should be investigated.

Despite the molecular-level investigations' limitations, they provided valuable, clinically relevant information. Molecular analysis detects only known resistance-encoding genes or mutations. Novel molecular mechanisms may not be detected unless they share a high level of similarity with a known gene. Moreover, the results of molecular tests may not fully correlate with the phenotypic variations that contribute to resistance. In addition, molecular analysis alone may not provide a complete understanding of the clinical outcomes and responses to antifungal treatments. It is crucial to consider the interplay of genetic modifications, phenotypic characteristics, and patient-related factors, allowing for a more comprehensive assessment of azole resistance [41, 42].

Beyond that, azole resistance could develop through mechanisms that molecular analysis may be unable to discover. Alterations in membrane permeability or changes in drug efflux pumps activities can contribute to azole resistance. Evaluating ergosterol levels, which are essential components of fungal cell membranes, can help assess the role of membrane-related modifications in resistance. Assessing efflux pump activity through efflux pump assays also provides important information on the functional aspects of resistance mechanisms. It helps determine whether resistant strains have increased efflux pump activity, leading to reduced intracellular drug accumulation [33, 43].

In the meantime, this is the first Egyptian study to investigate molecular mechanisms involved in azole resistance in clinical *C. tropicalis* isolates. The study has highlighted the contribution of the upregulated *CDR1* efflux pump and *the ERG11* gene substitutions, Y132F and G464S, as possible molecular mechanisms in fluconazole non-susceptibility in *C. tropicalis*.

Eventually, further studies are required to evaluate other molecular mechanisms involved in azole resistance, such as mutation and/or overexpression of ERG3, which is a C5 sterol desaturase enzyme that is involved in ergosterol biosynthesis and linked with azole resistance. In addition, evaluation of ERG11 gene expression levels and the possible substitutions in UPC2, which is the transcription factor that regulates the majority of ergosterol biosynthetic genes, as well as gene sequence analysis of MDR1, CDR1, and its transcription factor TAC1, may give a comprehensive overview on azole resistance in *C. tropicalis* clinical isolates.

### Supplementary Information

ESM 1 (DOCX 3.41 MB)

## Data Availability

The data presented in this study are available on request from the corresponding author.

## Abbreviations

*ABC*: ATP-binding cassette
*ATCC*: American type culture collection
*cDNA*: complementary deoxyribonucleic acid
*CDR*: *Candida* drug resistance
*CLSI*: Clinical and Laboratory Standards Institute
*DNA*: Deoxyribonucleic acid
*ECVs*: epidemiological cut-off values
*ICU*: Intensive care unit
*MDR*: Multiple drug resistance
*MFS*: major facilitator superfamily
*NAC*: non-*albicans Candida*
*RNA*: ribonucleic acid
*RT-qPCR*: reverse transcription quantitative polymerase chain reaction
*SDA*: Sabouraud dextrose agar
*SPSS*: Statistical Package for the Social Sciences
*SS-CBPs*: species-specific clinical breakpoints.
