# Supplementary material for: Analysis of CDR1 and MDR1 Gene Expression and ERG11 Substitutions in Clinical Candida tropicalis Isolates from Alexandria, Egypt
Source: Braz J Microbiol. 2023 Aug 22;54(4):2609–15. doi: 10.1007/s42770-023-01106-y (PMC10689625; doi:10.1007/s42770-023-01106-y)
Supplement: Supplementary file 1 — (DOCX 3.41 MB) [file 42770_2023_1106_MOESM1_ESM.docx]

|  | **Sample No.**  **Supplementary Table 1: Master table: Sample type and antifungal susceptibility prolife with MIC levels distribution among all isolated *C. tropicalis*.** | **Sample Type** | **Antifungal agent** | | | | | | | | | | | |
| --- | --- | --- | --- | --- | --- | --- | --- | --- | --- | --- | --- | --- | --- | --- |
| **#** |  |  | **Fluconazole** | | **Voriconazole** | | **Caspofungin** | | **Micafungin** | | **Amphotericin B** | | **Flucytosine** | |
|  |  |  | **MIC** | **Interp.** | **MIC** | **Interp.** | **MIC** | **Interp** | **MIC** | **Interp** | **MIC** | **Interp** | **MIC** | **Interp** |
| 1 | S1UR | Urine | 16 | R | 0.5 | SSD | ≤ 0.25 | S | ≤ 0.06 | S | 0.5 | S | ≤ 1 | S |
| 2 | S2US | Urine | ≤ 1 | S | ≤ 0.12 | S | ≤ 0.25 | S | ≤ 0.06 | S | 0.5 | S | ≤ 1 | S |
| 3 | S3RR | Resp. | 8 | R | 0.25 | SSD | ≤ 0.25 | S | ≤ 0.06 | S | 1 | S | ≤ 1 | S |
| 4 | S4UR | Urine | 32 | R | 0.5 | SSD | ≤ 0.25 | S | ≤ 0.06 | S | 0.5 | S | ≤ 1 | S |
| 5 | S5UR | Urine | ≥ 64 | R | 1 | R | ≤ 0.25 | S | ≤ 0.06 | S | ≤ 0.25 | S | ≤ 1 | S |
| 6 | S6US | Urine | ≤ 1 | S | ≤ 0.12 | S | ≤ 0.25 | S | ≤ 0.06 | S | 0.5 | S | ≤ 1 | S |
| 7 | S7UR | Urine | 32 | R | 0.5 | SSD | ≤ 0.25 | S | ≤ 0.06 | S | 0.5 | S | ≤ 1 | S |
| 8 | S8US | Urine | ≤ 1 | S | ≤ 0.12 | S | ≤ 0.25 | S | ≤ 0.06 | S | 0.5 | S | ≤ 1 | S |
| 9 | S9UR | Urine | 8 | R | 0.25 | SSD | ≤ 0.25 | S | ≤ 0.06 | S | 0.5 | S | ≤ 1 | S |
| 10 | S10BS | Blood | ≤ 1 | S | ≤ 0.12 | S | ≤ 0.25 | S | ≤ 0.06 | S | 0.5 | S | ≤ 1 | S |
| 11 | S11US | Urine | ≤ 1 | S | ≤ 0.12 | S | ≤ 0.25 | S | ≤ 0.06 | S | 0.5 | S | ≤ 1 | S |
| 12 | S12US | Urine | ≤ 1 | S | ≤ 0.12 | S | ≤ 0.25 | S | ≤ 0.06 | S | 0.5 | S | ≤ 1 | S |
| 13 | S13RR | Resp | 8 | R | ≤ 0.12 | S | ≤ 0.25 | S | ≤ 0.06 | S | 0.5 | S | ≤ 1 | S |
| 14 | S14RS | Resp | ≤ 1 | S | ≤ 0.12 | S | ≤ 0.25 | S | ≤ 0.06 | S | 0.5 | S | ≤ 1 | S |
| 15 | S15UR | Urine | 32 | R | 0.5 | SSD | ≤ 0.25 | S | ≤ 0.06 | S | 0.5 | S | ≤ 1 | S |
| 16 | S16SS | Skin | ≤ 1 | S | ≤ 0.12 | S | ≤ 0.25 | S | ≤ 0.06 | S | 0.5 | S | ≤ 1 | S |
| 17 | S17BS | Blood | ≤ 1 | S | ≤ 0.12 | S | ≤ 0.25 | S | ≤ 0.06 | S | 0.5 | S | ≤ 1 | S |
| 18 | S18RS | Resp. | ≤ 1 | S | ≤ 0.12 | S | ≤ 0.25 | S | ≤ 0.06 | S | 0.5 | S | ≤ 1 | S |
| 19 | S20US | Urine | ≤ 1 | S | ≤ 0.12 | S | ≤ 0.25 | S | ≤ 0.06 | S | 0.5 | S | ≤ 1 | S |
| 20 | S21US | Urine | ≤ 1 | S | ≤ 0.12 | S | ≤ 0.25 | S | ≤ 0.06 | S | ≤ 0.25 | S | ≤ 1 | S |
| 21 | S22SS | Skin | ≤ 1 | S | ≤ 0.12 | S | ≤ 0.25 | S | ≤ 0.06 | S | 0.5 | S | ≤ 1 | S |
| 22 | S23UR | Urine | 4 | SDD | 0.25 | SSD | ≤ 0.25 | S | ≤ 0.06 | S | 0.5 | S | ≤ 1 | S |
| 23 | S24UR | Urine | 8 | R | 0.25 | SSD | ≤ 0.25 | S | ≤ 0.06 | S | ≤ 0.25 | S | ≤ 1 | S |
| 24 | S25US | Urine | ≤ 1 | S | ≤ 0.12 | S | ≤ 0.25 | S | ≤ 0.06 | S | 0.5 | S | ≤ 1 | S |
| 25 | S26BS | Blood | ≤ 1 | S | ≤ 0.12 | S | ≤ 0.25 | S | ≤ 0.06 | S | ≤ 0.25 | S | ≤ 1 | S |
| 26 | S27UR | Urine | 8 | R | 0.25 | SSD | ≤ 0.25 | S | ≤ 0.06 | S | 0.5 | S | ≤ 1 | S |
| 27 | S28UR | Urine | 4 | SDD | 0.25 | SSD | ≤ 0.25 | S | ≤ 0.06 | S | ≤ 0.25 | S | ≤ 1 | S |
| 28 | S29RS | Resp | ≤ 1 | S | ≤ 0.12 | S | ≤ 0.25 | S | ≤ 0.06 | S | 0.5 | S | ≤ 1 | S |
| 29 | S30US | Urine | ≤ 1 | S | ≤ 0.12 | S | ≤ 0.25 | S | ≤ 0.06 | S | ≤ 0.25 | S | ≤ 1 | S |
| 30 | S31RS | Resp | ≤ 1 | S | ≤ 0.12 | S | ≤ 0.25 | S | ≤ 0.06 | S | ≤ 0.25 | S | ≤ 1 | S |
| 31 | S32US | Urine | ≤ 1 | S | ≤ 0.12 | S | ≤ 0.25 | S | ≤ 0.06 | S | ≤ 0.25 | S | ≤ 1 | S |
| 32 | S33BS | Blood | ≤ 1 | S | ≤ 0.12 | S | ≤ 0.25 | S | ≤ 0.06 | S | 0.5 | S | ≤ 1 | S |
| 33 | S34RS | Resp | ≤ 1 | S | ≤ 0.12 | S | ≤ 0.25 | S | ≤ 0.06 | S | 0.5 | S | ≤ 1 | S |
| 34 | S35US | Urine | ≤ 1 | S | ≤ 0.12 | S | ≤ 0.25 | S | ≤ 0.06 | S | ≤ 0.25 | S | ≤ 1 | S |
| 35 | S36US | Urine | ≤ 1 | S | ≤ 0.12 | S | ≤ 0.25 | S | ≤ 0.06 | S | 0.5 | S | ≤ 1 | S |
| 36 | S37BS | Blood | ≤ 1 | S | ≤ 0.12 | S | ≤ 0.25 | S | ≤ 0.06 | S | 0.5 | S | ≤ 1 | S |
| 37 | S39UR | Urine | 8 | R | 0.25 | SSD | ≤ 0.25 | S | ≤ 0.06 | S | 0.5 | S | ≤ 1 | S |
| 38 | S41UR | Urine | 8 | R | 0.25 | SSD | ≤ 0.25 | S | ≤ 0.06 | S | 0.5 | S | ≤ 1 | S |
| 39 | S47UR | Urine | 32 | R | 0.5 | SSD | ≤ 0.25 | S | ≤ 0.06 | S | ≤ 0.25 | S | ≤ 1 | S |
| 40 | S48UR | Urine | 4 | SDD | ≤ 0.12 | S | ≤ 0.25 | S | ≤ 0.06 | S | 0.5 | S | ≤ 1 | S |
| 41 | S52UR | Urine | ≥ 64 | R | 1 | R | ≤ 0.25 | S | ≤ 0.06 | S | 0.5 | S | ≤ 1 | S |
| 42 | S53UR | Urine | 32 | R | 0.5 | SSD | ≤ 0.25 | S | ≤ 0.06 | S | 0.5 | S | ≤ 1 | S |
| 43 | S54UR | Urine | 4 | SDD | 0.25 | SSD | ≤ 0.25 | S | ≤ 0.06 | S | 0.5 | S | ≤ 1 | S |
| 44 | S56UR | Urine | 8 | R | 0.25 | SSD | ≤ 0.25 | S | ≤ 0.06 | S | 0.5 | S | ≤ 1 | S |
| 45 | S57UR | Urine | 8 | R | 0.25 | SSD | ≤ 0.25 | S | ≤ 0.06 | S | 0.5 | S | ≤ 1 | S |
| 46 | S62UR | Urine | 8 | R | 0.25 | SSD | ≤ 0.25 | S | ≤ 0.06 | S | ≤ 0.25 | S | ≤ 1 | S |
| 47 | S64UR | Urine | 8 | R | 0.5 | SSD | ≤ 0.25 | S | ≤ 0.06 | S | 0.5 | S | ≤ 1 | S |
| 48 | S65UR | Urine | 32 | R | 2 | R | ≤ 0.25 | S | ≤ 0.06 | S | 0.5 | S | ≤ 1 | S |
| 49 | S67UR | Urine | 32 | R | 0.5 | SSD | ≤ 0.25 | S | ≤ 0.06 | S | ≤ 0.25 | S | ≤ 1 | S |
| 50 | S69UR | Urine | 8 | R | 0.25 | SSD | ≤ 0.25 | S | ≤ 0.06 | S | ≤ 0.25 | S | ≤ 1 | S |
| 51 | S71UR | Urine | 32 | R | 1 | R | ≤ 0.25 | S | ≤ 0.06 | S | 0.5 | S | ≤ 1 | S |
| 52 | S74UR | Urine | 32 | R | 2 | R | ≤ 0.25 | S | ≤ 0.06 | S | 0.5 | S | ≤ 1 | S |
| 53 | S75UR | Urine | 8 | R | 0.25 | SSD | ≤ 0.25 | S | ≤ 0.06 | S | 0.5 | S | ≤ 1 | S |
| 54 | S76RR | Resp | 32 | R | 0.5 | SSD | ≤ 0.25 | S | ≤ 0.06 | S | 0.5 | S | ≤ 1 | S |

**Supplementary Table 2: Expression levels of *CDR1* gene among fluconazole susceptible *C. tropicalis* isolates using ΔΔC_T_ method.**

| ***CDR1* gene in fluconazole susceptible isolates** | | | | | |
| --- | --- | --- | --- | --- | --- |
| **Sample** | ***CDR1* C_T_** | ***Actin* C_T_** | **ΔC_T_** | **ΔΔC_T_** | **Fold Change in Expression** |
| S2US | 16.719 | 16.164 | 0.555 | 0.350 | 0.785 |
| S6US | 16.926 | 15.804 | 1.122 | 0.918 | 0.529 |
| S8US | 16.420 | 15.656 | 0.764 | 0.559 | 0.679 |
| S10BS | 16.877 | 15.848 | 1.029 | 0.825 | 0.565 |
| S11US | 16.201 | 15.188 | 1.013 | 0.808 | 0.571 |
| S12US | 18.368 | 18.231 | 0.137 | -0.067 | 1.048 |
| S14RS | 16.660 | 16.254 | 0.406 | 0.201 | 0.870 |
| S16SS | 18.466 | 18.334 | 0.133 | -0.072 | 1.051 |
| S17BS | 15.967 | 16.607 | -0.640 | -0.844 | 1.796 |
| S18RS | 15.451 | 15.779 | -0.328 | -0.533 | 1.447 |
| S20US | 14.807 | 14.700 | 0.107 | -0.098 | 1.070 |
| S21US | 16.595 | 16.158 | 0.437 | 0.233 | 0.851 |
| S22SS | 16.891 | 15.942 | 0.950 | 0.745 | 0.597 |
| S25US | 16.383 | 15.470 | 0.913 | 0.709 | 0.612 |
| S26BS | 18.464 | 18.185 | 0.279 | 0.074 | 0.950 |
| S29RS | 16.213 | 16.471 | -0.258 | -0.463 | 1.378 |
| S30US | 15.494 | 15.742 | -0.248 | -0.452 | 1.368 |
| S31RS | 14.814 | 14.672 | 0.142 | -0.062 | 1.044 |
| S32US | 18.268 | 18.533 | -0.265 | -0.470 | 1.385 |
| S33BS | 16.938 | 17.510 | -0.572 | -0.776 | 1.713 |
| S34RS | 16.129 | 16.575 | -0.446 | -0.651 | 1.570 |
| S35US | 14.828 | 14.695 | 0.133 | -0.072 | 1.051 |
| S36US | 18.390 | 18.283 | 0.107 | -0.097 | 1.070 |
| S37BS | 16.998 | 17.558 | -0.560 | -0.764 | 1.698 |

**Supplementary Table 3: Expression levels of *CDR1* gene among fluconazole non-susceptible *C. tropicalis* isolates using ΔΔC_T_ method.**

| ***CDR1* gene in fluconazole non-susceptible isolates** | | | | | |
| --- | --- | --- | --- | --- | --- |
| **Sample** | ***CDR1* C_T_** | ***Actin* C_T_** | **ΔC_T_** | **ΔΔC_T_** | **Fold Change in Expression** |
| S1UR | 15.530 | 15.484 | 0.046 | -0.158 | 1.116 |
| S3RR | 29.546 | 16.672 | 12.875 | 12.670 | 0.000 |
| S4UR | 16.240 | 17.212 | -0.973 | -1.177 | 2.261 |
| S5UR | 16.076 | 16.172 | -0.096 | -0.301 | 1.232 |
| S7UR | 17.681 | 15.573 | 2.108 | 1.903 | 0.267 |
| S9UR | 15.561 | 15.441 | 0.120 | -0.085 | 1.060 |
| S13RR | 15.418 | 15.290 | 0.128 | -0.077 | 1.055 |
| S15UR | 15.988 | 15.260 | 0.728 | 0.524 | 0.696 |
| S23UR | 15.833 | 15.765 | 0.068 | -0.136 | 1.099 |
| S24UR | 17.080 | 18.718 | -1.638 | -1.842 | 3.586 |
| S27UR | 17.149 | 19.089 | -1.940 | -2.144 | 4.420 |
| S28UR | 16.823 | 18.157 | -1.334 | -1.539 | 2.906 |
| S39UR | 15.802 | 18.640 | -2.837 | -3.042 | 8.236 |
| S41UR | 17.021 | 17.252 | -0.231 | -0.435 | 1.352 |
| S47UR | 17.242 | 17.447 | -0.205 | -0.410 | 1.328 |
| S48UR | 17.389 | 15.955 | 1.434 | -1.001 | 2.001 |
| S52UR | 16.077 | 16.241 | -0.165 | -0.369 | 1.292 |
| S53UR | 15.125 | 15.513 | -0.388 | -0.593 | 1.508 |
| S54UR | 17.236 | 16.491 | 0.744 | 0.539 | 0.688 |
| S56UR | 15.726 | 16.474 | -0.748 | -0.953 | 1.936 |
| S57UR | 16.447 | 18.186 | -1.739 | -1.943 | 3.846 |
| S62UR | 15.129 | 15.711 | -0.582 | -0.787 | 1.725 |
| S64UR | 15.876 | 16.807 | -0.931 | -1.135 | 2.197 |
| S65UR | 21.505 | 24.393 | -2.888 | -3.093 | 8.531 |
| S67UR | 19.011 | 19.981 | -0.970 | -1.175 | 2.258 |
| S69UR | 15.704 | 16.003 | -0.299 | -0.504 | 1.418 |
| S71UR | 15.263 | 16.335 | -1.073 | -1.277 | 2.424 |
| S74UR | 17.032 | 16.868 | 0.165 | -0.040 | 1.028 |
| S75UR | 18.389 | 18.789 | -0.400 | -0.605 | 1.520 |
| S76RR | 18.606 | 19.769 | -1.163 | -1.368 | 2.580 |

**Supplementary Table 4: Expression levels of *MDR1* gene among fluconazole susceptible *C. tropicalis* isolates using ΔΔC_T_ method.**

| ***MDR1* gene in fluconazole susceptible isolates** | | | | | |
| --- | --- | --- | --- | --- | --- |
| **Sample** | ***MDR1* C_T_** | ***Actin* C_T_** | **Δ C_T_** | **ΔΔC_T_** | **Fold Change in Expression** |
| S2US | 20.309 | 16.164 | 4.145 | -1.423 | 2.681 |
| S6US | 24.246 | 15.804 | 8.442 | 2.875 | 0.136 |
| S8US | 19.934 | 15.656 | 4.278 | -1.290 | 2.446 |
| S10BS | 24.11 | 15.85 | 8.267 | 2.699 | 0.154 |
| S11US | 19.417 | 15.188 | 4.229 | -1.339 | 2.530 |
| S12US | 22.564 | 18.231 | 4.333 | -1.235 | 2.353 |
| S14RS | 20.63 | 16.25 | 4.376 | -1.19 | 2.284 |
| S16SS | 22.753 | 18.334 | 4.419 | -1.149 | 2.217 |
| S17BS | 21.803 | 16.607 | 5.196 | -0.372 | 1.294 |
| S18RS | 21.574 | 15.779 | 5.795 | 0.227 | 0.854 |
| S20US | 20.609 | 14.700 | 5.909 | 0.341 | 0.790 |
| S21US | 20.357 | 16.158 | 4.199 | -1.369 | 2.582 |
| S22SS | 23.598 | 15.942 | 7.656 | 2.088 | 0.235 |
| S25US | 19.852 | 15.470 | 4.382 | -1.186 | 2.275 |
| S26BS | 22.442 | 18.185 | 4.257 | -1.311 | 2.481 |
| S29RS | 21.73 | 16.47 | 5.259 | -0.31 | 1.239 |
| S30US | 21.18 | 15.74 | 5.442 | -0.13 | 1.091 |
| S31RS | 20.71 | 14.67 | 6.041 | 0.473 | 0.720 |
| S32US | 25.1 | 18.53 | 6.564 | 0.996 | 0.501 |
| S33BS | 23.67 | 17.51 | 6.162 | 0.595 | 0.662 |
| S34RS | 21.844 | 16.575 | 5.269 | -0.299 | 1.230 |
| S35US | 20.677 | 14.695 | 5.983 | 0.415 | 0.750 |
| S36US | 24.751 | 18.283 | 6.468 | 0.901 | 0.536 |
| S37BS | 24.113 | 17.558 | 6.555 | 0.988 | 0.504 |

**Supplementary Table 5: Expression levels of *MDR1* gene among fluconazole non-susceptible *C. tropicalis* isolates using ΔΔC_T_ method.**

| ***MDR1* gene in fluconazole non-susceptible isolates** | | | | | |
| --- | --- | --- | --- | --- | --- |
| **Sample** | ***MDR1* C_T_** | ***Actin* C_T_** | **ΔC_T_** | **ΔΔC_T_** | **Fold Change in Expression** |
| S1UR | 21.498 | 15.484 | 6.014 | 0.446 | 0.734 |
| S3RR | 23.154 | 16.672 | 6.482 | 0.915 | 0.530 |
| S4UR | 21.491 | 17.212 | 4.279 | -1.289 | 2.443 |
| S5UR | 21.224 | 16.172 | 5.052 | -0.516 | 1.430 |
| S7UR | 21.191 | 15.573 | 5.618 | 0.050 | 0.966 |
| S9UR | 20.618 | 15.441 | 5.177 | -0.390 | 1.311 |
| S13RR | 20.542 | 15.290 | 5.251 | -0.316 | 1.245 |
| S15UR | 22.325 | 15.260 | 7.066 | 1.498 | 0.354 |
| S23UR | 21.898 | 15.765 | 6.133 | 0.565 | 0.676 |
| S24UR | 23.325 | 18.718 | 4.607 | -0.961 | 1.947 |
| S27UR | 23.933 | 19.089 | 4.844 | -0.724 | 1.652 |
| S28UR | 20.592 | 18.157 | 2.435 | -3.133 | 8.770 |
| S39UR | 21.367 | 18.640 | 2.727 | -2.841 | 7.164 |
| S41UR | 21.442 | 17.252 | 4.190 | -1.378 | 2.599 |
| S47UR | 26.298 | 17.447 | 8.851 | 3.283 | 0.103 |
| S48UR | 21.771 | 15.955 | 5.816 | 0.248 | 0.842 |
| S52UR | 24.371 | 16.241 | 8.129 | 2.562 | 0.169 |
| S53UR | 22.596 | 15.513 | 7.083 | 1.516 | 0.350 |
| S54UR | 24.183 | 16.491 | 7.691 | 2.123 | 0.229 |
| S56UR | 22.223 | 16.474 | 5.749 | 0.181 | 0.882 |
| S57UR | 24.102 | 18.186 | 5.916 | 0.349 | 0.785 |
| S62UR | 21.228 | 15.711 | 5.517 | -0.050 | 1.035 |
| S64UR | 23.050 | 16.807 | 6.243 | 0.675 | 0.626 |
| S65UR | 26.691 | 24.393 | 2.298 | -3.270 | 9.645 |
| S67UR | 24.452 | 19.981 | 4.471 | -1.097 | 2.139 |
| S69UR | 21.537 | 16.003 | 5.533 | -0.034 | 1.024 |
| S71UR | 22.547 | 16.335 | 6.211 | 0.644 | 0.640 |
| S74UR | 24.500 | 16.868 | 7.632 | 2.064 | 0.239 |
| S75UR | 25.618 | 18.789 | 6.829 | 1.261 | 0.417 |
| S76RR | 24.625 | 19.769 | 4.856 | -0.712 | 1.638 |


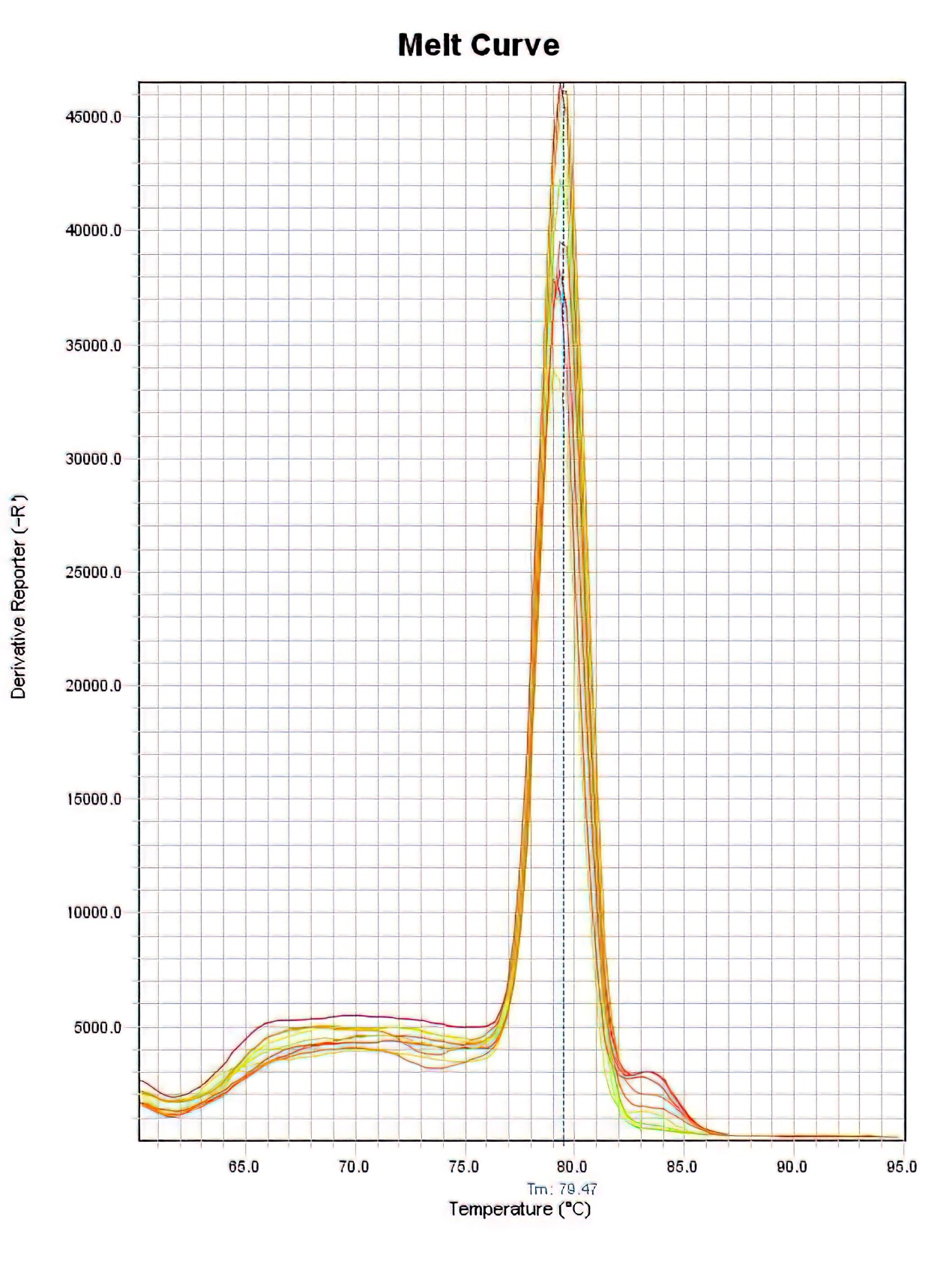
 **Supplementary Figure 1: Melt curve analysis of *CDR1* gene PCR reaction confirming the specificity of the reaction.**


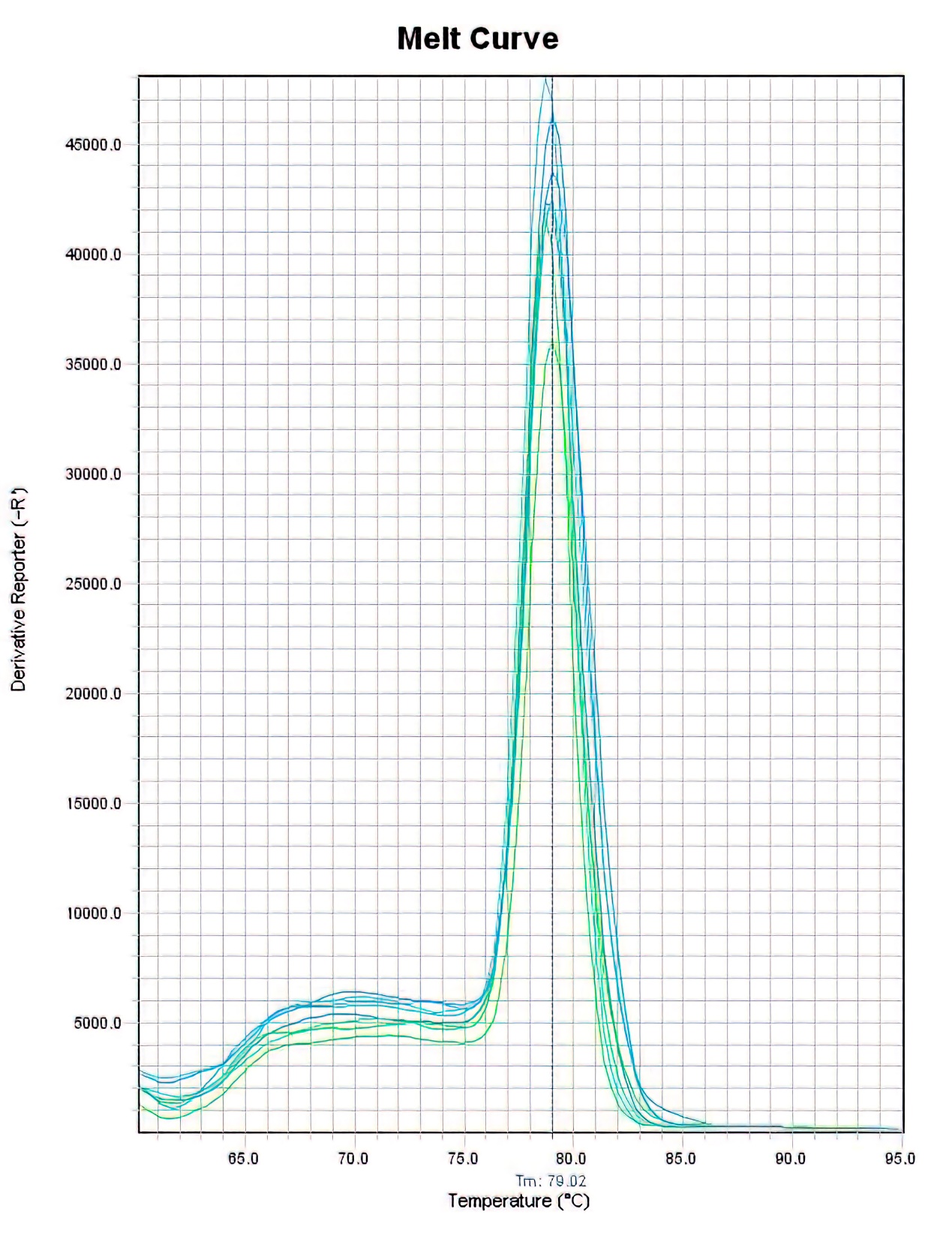


**Supplementary Figure 2: Melt curve analysis of *MDR* gene PCR reaction confirming the specificity of the reaction**


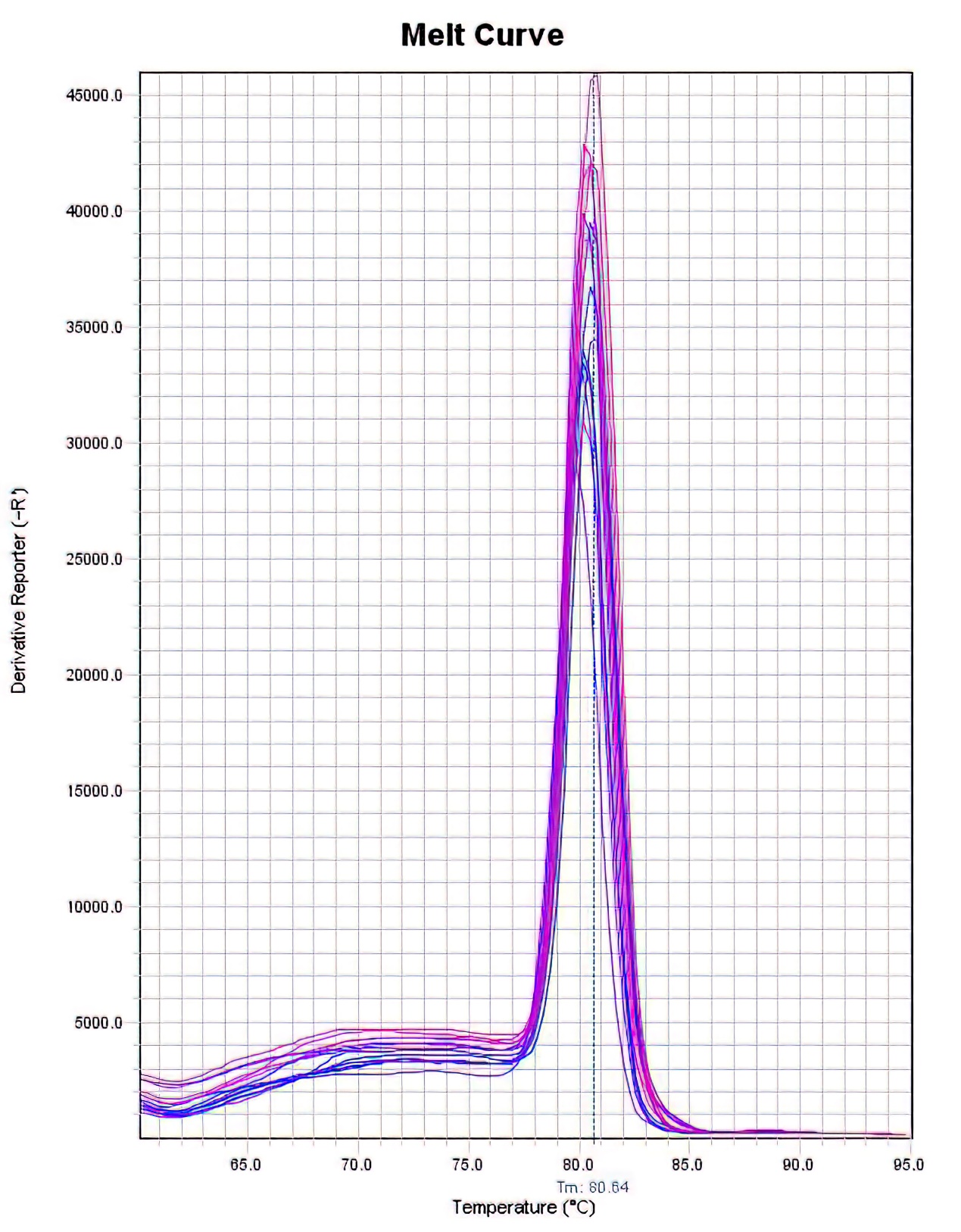


**Supplementary Figure 3: Melt curve analysis of Actin gene PCR reaction confirming the specificity of the reaction**


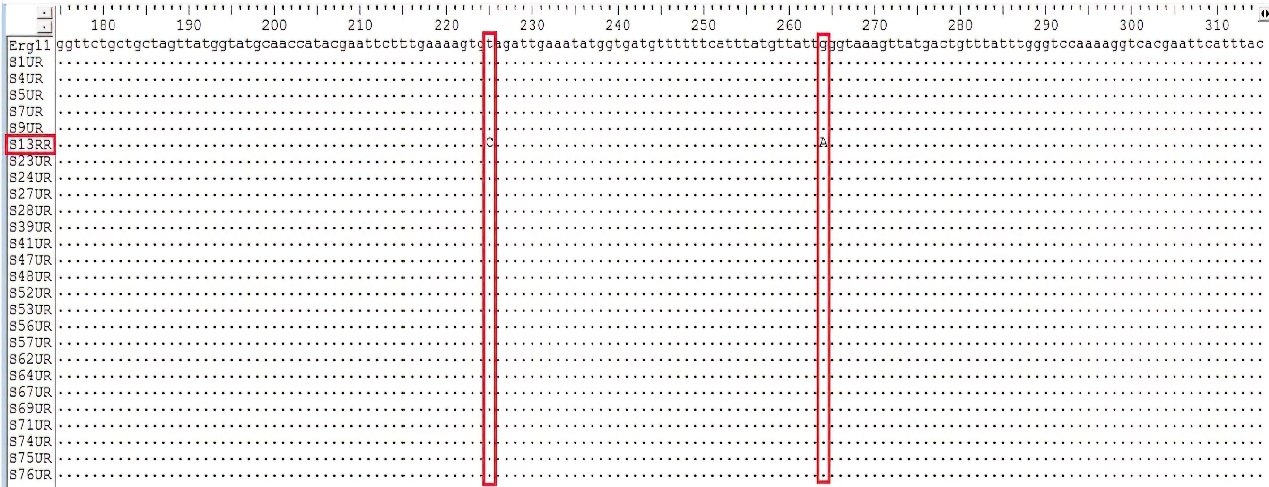


**Supplementary Figure 4: *ERG11* gene sequence alignment of *C. tropicalis* isolates and GenBank strain *(C. tropicalis* ATCC 750) demonstrating the point mutations T225C and G264A.**


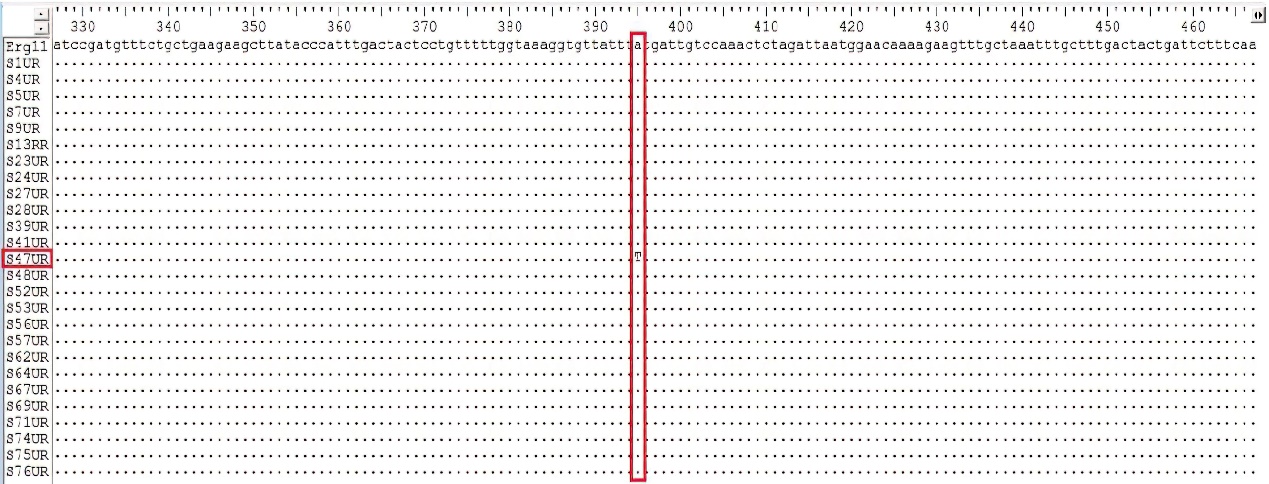


**Supplementary Figure 5: *ERG11* gene sequence alignment of *C. tropicalis* isolates and GenBank strain *(C. tropicalis* ATCC 750) demonstrating the point mutation A395T.**


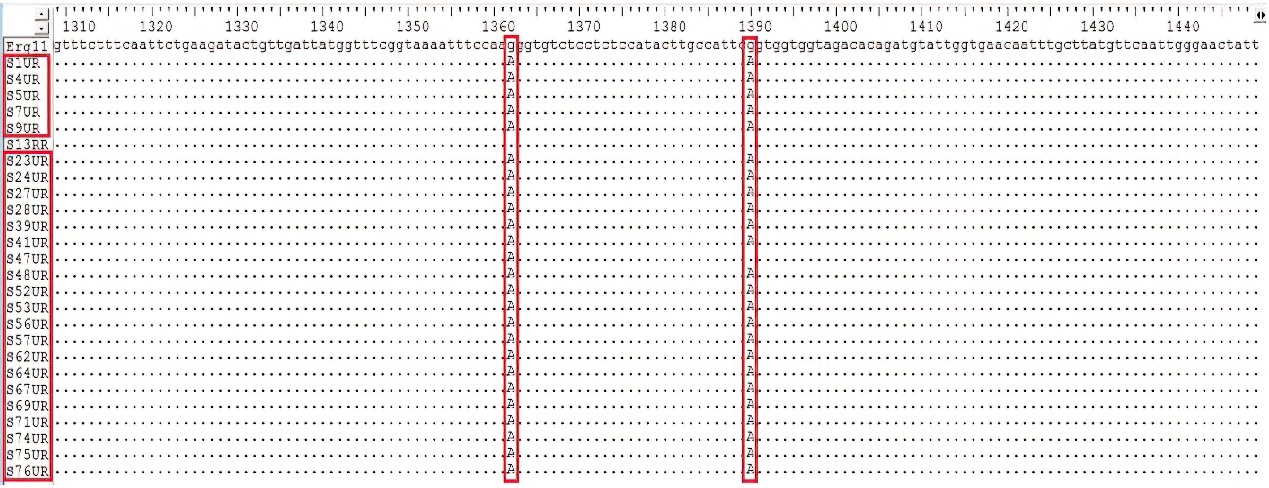


**Supplementary Figure 6: *ERG11* gene sequence alignment of *C. tropicalis* isolates and GenBank strain (*C. tropicalis* ATCC 750) demonstrating the point mutations G1362A and G1390A.**


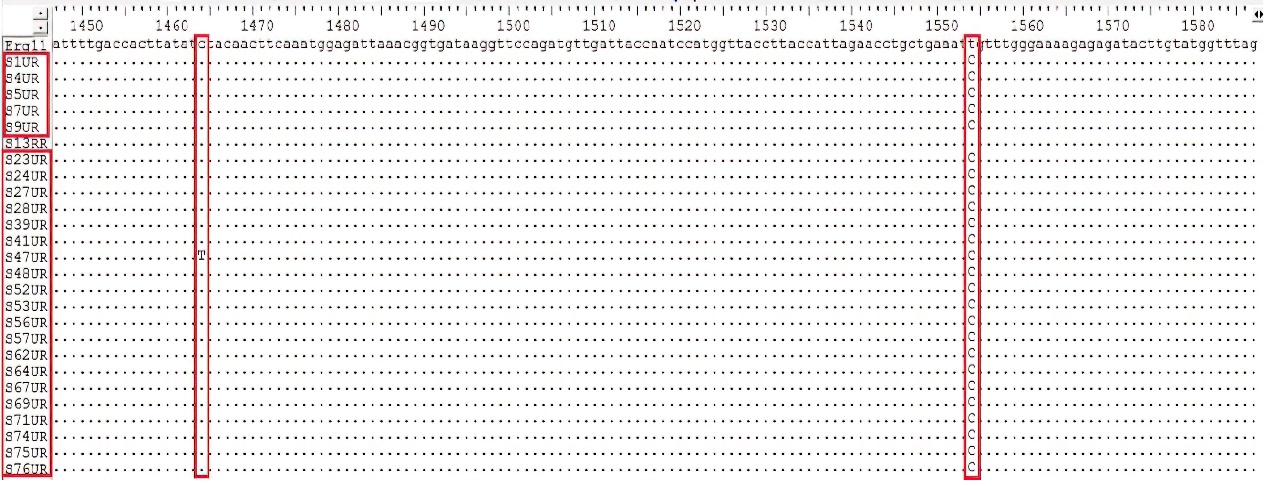


**Supplementary Figure 7: *ERG11* gene sequence alignment of *C. tropicalis* isolates and GenBank strain (*C. tropicalis* ATCC 750) demonstrating the point mutations C1464T and T1554C.**


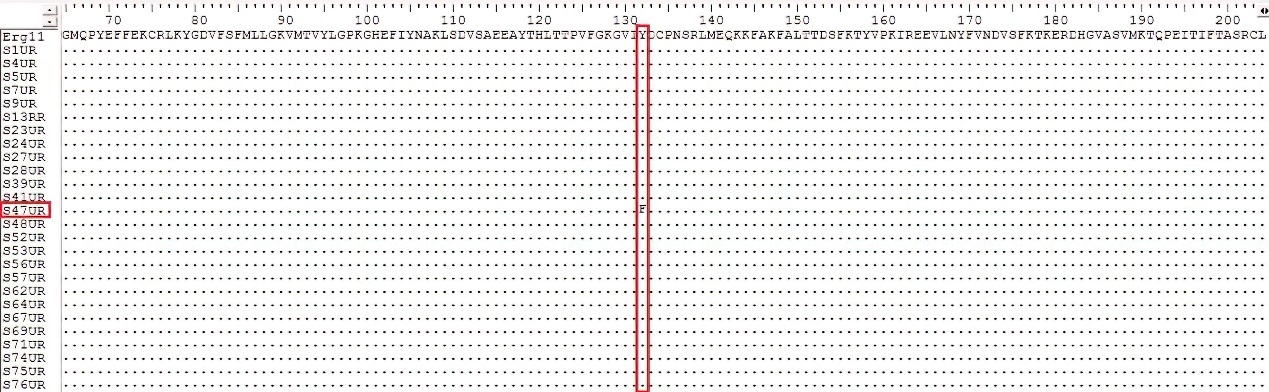


**Supplementary Figure 8: Alignment of the amino acid residues of the Erg11p sequences obtained for the isolates characterized in this study with a reference sequence retrieved from the GenBank strain (*C. tropicalis* ATCC 750) demonstrating Y132F substitution.**


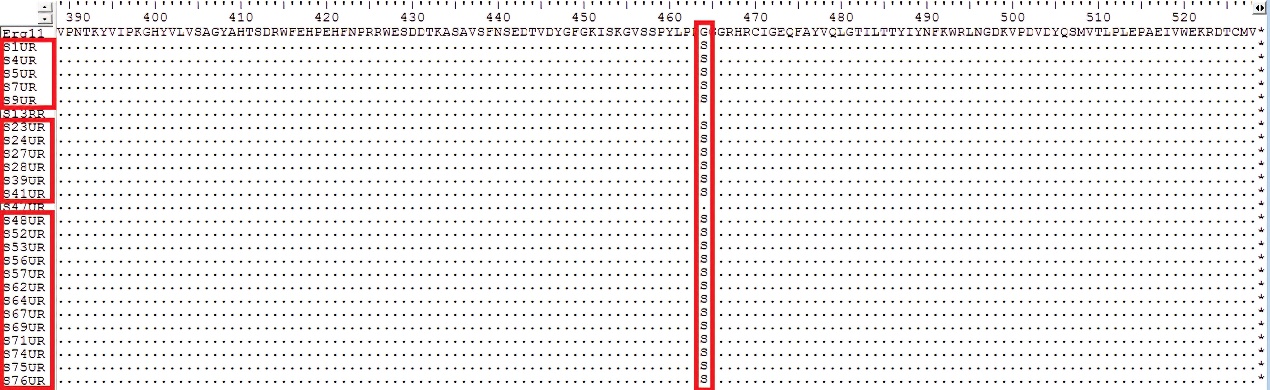


**Supplementary Figure 9: Alignment of the amino acid residues of the Erg11p sequences obtained for the isolates characterized in this study with a reference sequence retrieved from the GenBank strain (*C. tropicalis* ATCC 750) demonstrating G464S substitution.**
